# The role and mechanism of transforming growth factor beta 3 in human myocardial infarction‐induced myocardial fibrosis

**DOI:** 10.1111/jcmm.14313

**Published:** 2019-04-14

**Authors:** Ke Xue, Jun Zhang, Cong Li, Jing Li, Cong Wang, Qingqing Zhang, Xianlu Chen, Xiaotang Yu, Lei Sun, Xiao Yu

**Affiliations:** ^1^ Department of Pathology and Forensic Medicine, College of Basic Medical Sciences Dalian Medical University Dalian China

**Keywords:** cardiac fibroblasts, myocardial fibrosis, myocardial infarction, smad7, TGFβ3

## Abstract

Transforming growth factor beta (TGFβ) plays a crucial role in tissue fibrosis. A number of studies have shown that TGFβ3 significantly attenuated tissue fibrosis. However, the mechanism involved in this effect is poorly understood. In this study we found that the expression level of TGFβ3 was higher in human myocardial infarction (MI) tissues than in normal tissues, and interestingly, it increased with the development of fibrosis post‐myocardial infarction (post‐MI). In vitro, human cardiac fibroblasts (CFs) were incubated with angiotensin II (Ang II) to mimic the ischaemic myocardium microenvironment and used to investigate the anti‐fibrotic mechanism of TGFβ3. Then, fibrosis‐related proteins were detected by Western blot. It was revealed that TGFβ3 up‐regulation attenuated the proliferation, migration of human CFs and the expression of collagens, which are the main contributors to fibrosis, promoted the phenotype shift and the cross‐linking of collagens. Importantly, the expression of collagens was higher in the si‐smad7 groups than in the control groups, while silencing smad7 increased the phosphorylation level of the TGFβ/smad signalling pathway. Collectively, these results indicated that TGFβ3 inhibited fibrosis via the TGFβ/smad signalling pathway, possibly attributable to the regulation of smad7, and that TGFβ3 might serve as a potential therapeutic target for myocardial fibrosis post‐MI.

## 1 INTRODUCTION

Myocardial remodelling after myocardial infarction (MI) is a major factor that accelerates heart failure. Several pathophysiological processes are involved in MI, including inflammation, cell apoptosis, angiogenesis, hypertrophy, extracellular matrix (ECM) synthesis and deposition^1^ and cross‐linking of collagens.^2^ Excessive ECM deposition results in cardiac fibrosis, which leads to attenuated ventricular compliance, cardiac dysfunction and eventually heart failure.^3^ The critical phase of these responses depends on the alteration of the cardiac microenvironment, which includes inflammation changes, the proliferation and migration of nonmyocytes.^4^

Cardiac fibroblasts (CFs) play a critical role in maintaining normal cardiac function and in cardiac remodelling during pathological conditions, such as MI and hypertension. These cells have numerous functions, including ECM synthesis and deposition, cell‐cell communication with myocytes and cell‐cell signalling with other fibroblasts and endothelial cells. In the early phase of MI, CFs, characterized by their fundamental contribution to the cardiac response to various forms of injury, accumulate in the infarcted area in response to cytokine recruitment.^5^ In the maturation phase, the CFs secrete collagens and other ECM proteins to maintain the stability of the scar.^6^ The phenotype of CFs changes to that of myofibroblasts. Thus, regulating the synthesis, deposition and cross‐linking of collagens is a key step in improving post‐MI prognosis.

Transforming growth factor beta (TGFβ), a multifunctional peptide superfamily, regulates cell growth, differentiation and influence the action of the cellular receptors.^7^ Only the TGFβ1, TGFβ2 and TGFβ3 isoforms have been isolated from human sources.^8^ A number of studies showed that this superfamily affected a variety of biological processes in tissue fibrosis,^7^ of which TGFβ1 and TGFβ2 were viewed as critical molecular factors that drive the formation of fibrosis accompanying many disease states; nevertheless, TGFβ3 down‐regulated scarring and fibrosis in vivo under certain experimental conditions.^9^

The expression of TGFβ3, which is high in the embryonic wound microenvironment,^10^ was increased predominantly and persistently in infarcted areas in rats.^11^ Thus, TGFβ3 appeared to be a very promising candidate for the prognosis of MI. However, the pathological roles of TGFβ3 in post‐MI fibrosis and the molecular mechanisms underlying myocardial fibroblasts proliferation, migration, phenotype shift and function remain poorly understood.^7^ Many clinical data indicated that Angiotensin II (Ang II) played an important role in myocardial remodelling after MI.^12^, ^13^ Cardiac Ang II levels were quickly elevated after injury which stimulated proliferation, migration, phenotype shift and collagen synthesis in CFs.^14^ Candesartanan, an Ang II AT1 receptor blocker, led to an up‐regulation of TGFβ3 expression in cortical and hippocampal astrocytes in mice.^15^ Considering the potential anti‐fibrotic role of TGFβ3 in the context of tissue repair and remodelling, and the well‐known involvement of Ang II in fibroblast activation and matrix deposition, we hypothesized that TGFβ3 might have certain effects on Ang II‐induced myocardial fibrosis.

TGFβ exerts its biological function by smad signal transduction. Smad proteins typically consist of two domains. The amino Mad homology (MH) 1 domain has DNA binding capabilities, while the carboxyl MH2 domain has been shown to mediate interactions with a variety of proteins. In the nucleus, the smad complex binds to promoters or enhancers of TGFβ target genes in order to induce cell‐specific transcriptional programs.^16^

In this study, we measured the expression levels of TGFβ1, TGFβ2 and TGFβ3 in human normal myocardial areas, MI areas and in serum samples. Subsequently, we applied exogenous and endogenous TGFβ3 to human CFs to analyse the effects of TGFβ3 on cell proliferation, migration, phenotype shift, collagen synthesis and cross‐linking. To explore the possible molecular mechanism by which TGFβ3 regulates fibrotic potential, we applied Ang II‐induced medium to human CFs and analysed the relative levels of collagen synthesis, cross‐linking, TGFβ/smad molecules and their phosphorylation. Finally, we examined the role of smad7 in the TGFβ/smad signalling pathway.

## 2 METHODS AND MATERIALS

### 2.1 Tissue and cell culture

Control samples (n = 6), the early phase of MI (n = 6) and the late phase of MI (n = 6) experimental samples, which were obtained from the anterior wall of the left ventricle near the apex of the heart in MI patients of 30 to 70 years, and their serum specimens were collected within 48 hours after death by the Department of Pathology and Forensic Medicine, Dalian Medical University. All procedures concerning human samples conformed to the principles outlined in the Declaration of Helsinki and were approved by Dalian Medical University. Material care was reviewed and approved by the Institutional Ethics Committee. Human CFs (BIOLEAF) (passages 1‐20) were cultured in culture flasks. After being washed twice with serum‐free medium, the cells were incubated with serum‐containing 10% Dulbecco's modified Eagle's medium/F12 (DMEM/F12) medium in a humidified incubator at 37°C and 5%CO_2_. Ang II(Proteintech), recombinant transforming growth factor beta (rTGFβ3) protein (Proteintech) and other transfection reagents (GenePharma) were used to treat cells according to the experimental design.

### 2.2 Haematoxylin‐eosin(HE) staining

Tissues were fixed with 4% para‐formaldehyde, embedded in paraffin and cross‐sectioned (4 µm). An HE staining was performed to distinguish the normal tissue, the early phase and the late phase tissues of the MI following the manufacturer's instructions (ZSGB‐BIO). Marginal contraction band necrosis and neutrophilic infiltration are detected within 3 days of infarction. By 10 to 14 days after MI, collagen deposition becomes detectable. Increased collagen deposition with decreased cellularity indicated more than 2 weeks after MI.^17^ We grouped the cases within 3 days as the early phase of MI and the cases which more than 2 weeks as the late one. The sections were observed under an optical microscope. The tissues were dyed pink to indicate the cytoplasm and blue‐violet to indicate the cell nuclei.

### 2.3 Masson staining

Paraffin slides (4 µm) were used in Masson staining, which was performed to distinguish the normal tissue, the early phase and the late phase tissues of the MI following the manufacturer's instructions (Solarbio). The silk‐like fibres indicated the early phase of MI and the appearance of collagen deposition indicated the late phase of MI. The muscle fibres appeared red and the collagen fibres appeared blue.

### 2.4 Immunohistochemical staining

To detect the expression and distribution of TGFβ1 (1:100; Proteintech), TGFβ2 (1:100; Proteintech) and TGFβ3 (1:100; Proteintech), the slides of infarct tissues were immunohistochemically stained with the relevant antibodies following the manufacturer's instructions (ZSGB‐BIO) and the results were qualitatively analysed statistically in paraffin slides (4 µm). The slides were quantified by randomly choosing five fields in the infarcted area from each section. The sections were observed under an optical microscope. TGFβ1, TGFβ2 and TGFβ3 were visualized with 3,3‐diaminobenzidine (DAB), the chromogenic substrate for peroxidase. The brown‐yellow tissues indicated positive staining.

### 2.5 ELISA

An ELISA was performed on the serum samples to measure the levels of TGFβ1(Elabscience), TGFβ2(Elabscience) and TGFβ3(Elabscience) according to the manufacturer's instructions. Human serum samples were derived from autopsy cases. All procedures concerning human samples conformed to the principles outlined in the Declaration of Helsinki and were approved by Dalian Medical University. Material care was reviewed and approved by the Institutional Ethics Committee.

### 2.6 Immunofluorescence staining

An immunofluorescence assay was performed to detect the expression and distribution of α‐smooth muscle actin (α‐SMA; 1:200; Proteintech) and vimentin (1:200; Proteintech) as a control in human CFs to detect phenotype shifts in human CFs. Moreover, immunofluorescence staining using discoidin domain receptor 2 (DDR2; 1:200; Proteintech) and fibroblast‐specific protein 1 (FSP‐1; 1:200; Proteintech) antibodies was performed to mark the CFs, and the nuclei were counterstained with 0.5 µg/mL 4′,6‐diamidino‐2‐phenylindole (DAPI; Solarbio). Staining was analysed using an immunofluorescence microscope (Olympus).

### 2.7 Cell proliferation assay

Human CFs was seeded in 96‐well plates at a density of 2000 cells/well, and proliferation was evaluated using Cell Counting Kit‐8 (CCK‐8; Trans) assay according to the experimental proposal. Ten microliters of CCK‐8 solution was added to each well, and the cells were incubated for another 2 hours in a humidified incubator. Optical density was measured at 450 nm using a microplate reader (Thermo Scientific). Five replicate wells were set up for each group, and three independently repeated experiments were performed.

### 2.8 Cell toxicity assay

Human CFs was seeded in 96‐well plates at a density of 2000 cells/well, and toxicity was evaluated using MTT (Solarbio) assays according to the experimental proposal. Twenty microlitres of MTT was added to each well, and the cells were incubated for another 4 hours in a humidified incubator. The formazan crystals were solubilized with 20 μL DMSO and then optical density was measured at 490, 570 and 630 nm using a microplate reader (Thermo Scientific). Five replicate wells were set up for each group, and three independently repeated experiments were performed.

### 2.9 Western blot analysis

Cells were lysed, and proteins in the supernatant extracts were quantified using a BCA Protein Assay Kit (Beyotime). Total cell lysates containing 50 µg of protein were separated using sodium dodecyl sulfate‐polyacrylamide gel electrophoresis (SDS‐PAGE) and transferred onto nitrocellulose (NC) membranes (PALL). After blocking with 5% non‐fat dry milk in Tris‐buffered saline‐Tween‐20 (TBST) for 2 hours, the membranes were incubated with primary antibodies [the glyceraldehyde‐3‐phosphate dehydrogenase (GAPDH) antibody diluted at 1:3000; collagen I, collagen III, lysyl oxidase (LOX), osteopontin (OPN), vimentin, α‐SMA, smad2, p‐smad2, smad3, p‐smad3, smad4, smad7 and p‐smad7 antibodies diluted at 1:2000 or 1:1000] at 4°C overnight. The collagen I, collagen III, p‐smad2, p‐smad3 and p‐smad7 antibodies were purchased from Abcam, and the GAPDH, β‐tubulin, smad2, smad3, smad4, smad7, LOX, OPN, vimentin and α‐SMA antibodies were purchased from Proteintech. After three washes with TBST, the membranes were incubated with the corresponding horseradish peroxidise (HRP)‐conjugated secondary antibody (1:5000, GE, HyClone) at 37°C for 2 hours. The protein bands were visualized with enhanced chemiluminescence (ECL; Advansta) and detected using a ChemiDoc^TM^ MP imaging system (BIO‐RAD). The protein bands were then scanned using Image Lab^TM^ Software Version 4.1.

### 2.10 Cell transfection

TGFβ3 full length (pEX4‐TGFβ3) plasmid, TGFβ3 small interfering RNA (siRNA‐TGFβ3‐homo‐1423), smad7 small interfering RNA (siRNA‐smad7‐homo‐1426) and a non‐targeting sequence (negative control, NC) were synthesized by GenePharma. The sequences of the siRNAs were shown in Table 1 Cells were seeded into six‐well plates (Corning) until their growth density was 50%‐70% and then transfected with Lipofectamine 2000 reagent (Thermo Scientific) and the indicated siRNA or plasmid with serum‐free DMEM/F12. After 6 hours of incubation, the medium was replaced with DMEM/F12 medium with 10% foetal calf serum (FBS). The total time for proliferation and migration assays was 24 hours in which cells were seeded in the 96‐well plates for another 18 hours later. Over‐expression, silencing efficiencies were analysed using reverse transcription and quantitative polymerase chain reaction (RT‐qPCR) 24 hours after the transfection and Western blot assay 48 hours after the transfection.

2.11

**Table 1:** siRNA sequences used in the study

Genes	Primers sequence
si‐TGFβ3	Forward：GGAAUACUAUGCCAAAGAATT
	Reverse：UUCUUUGGCAUAGUAUUCCTT
si‐smad7	Forward：CCAAUGACCACGAGUUUAUTT
	Reverse：AUAAACUCGUGGUCAUUGGTT
Si‐NC	Forward：UUCUCCGAACGUGUCACGUTT
	Reverse：ACGUGACACGUUCGGAGAATT

### 2.12 Cell migration assay

Human CFs migration in vitro was assayed using a transwell chamber with a polycarbonic membrane (6.5 mm diameter and 8 µm pore size; Corning). Human CFs were trypsinized and re‐suspended in serum‐free medium at a density of 5 × 10^4^ cells/mL. Two hundred microlitre of the no‐transfected or transfected cell suspension was added to the upper chamber, and 600 µL of serum‐free DMEM/F12 supplemented with different concentrations of TGFβ3 (0, 5, 10 or 50 ng/mL) or Ang II of 100 nmol/L was added to the lower chamber according to the experimental requirements. Non‐migrating cells on the top surface of the membrane were removed with cotton swabs. The cells that migrated to the lower surface of the membrane were fixed with 4% methanol and stained with 10% Giemsa (Solarbio). The cell number was measured within five randomly chosen fields at 200 × magnification, and the average number was calculated with ImageJ.

### 2.13 RT‐qPCR

Total RNA was extracted from cultured human CFs with Trizol reagent (Thermo Scientific). The RNA concentration and quality were determined according to the 260/280 nm ratio measured using a NanoDrop spectrophotometer (ND‐100, Thermo Scientific). Total cDNA was synthesized by TransScript One‐Step gDNA Removal and cDNA Synthesis SuperMix (Trans). The mRNA expression of target genes was quantified by RT‐qPCR using TransStart Top Green qPCR SuperMix (Trans). The GAPDH housekeeping gene was used as a control for smad7 and TGFβ3. The relative expression quantity was calculated by 2^‐△△Ct^. The sequences of the primers used in RT‐qPCR were listed in Table 2.

**Table 2:** Primer sequences used in the study

Genes	Primers sequence
TGFβ3	Forward：TGCGCCCCCTCTACATTG
	Reverse：GGTTCGTGGACCCATTTCC
Smad7	Forward：TGCCTTCCTCCGCTGAAAC
	Reverse：CACAGTAGAGCCTCCCCACTCT
GAPDH	Forward：GTGGAAGGACTCATGACCACAGT
	Reverse：GGAAGGCCATGCCAGTGA

### 2.14 Statistical analysis

The results are expressed as the mean ± standard error (SEM). Inter‐group comparisons were performed using a one‐way analysis of variance for results from at least three independent experiments. Differences were considered statistically significant when *P* < 0.05. All statistical analyses were performed using GraphPad 5.0 statistical software (IBM).

## 3 RESULTS

### 3.1 Human MI samples exhibited higher TGFβ3 levels than normal samples

**Figure 1:**
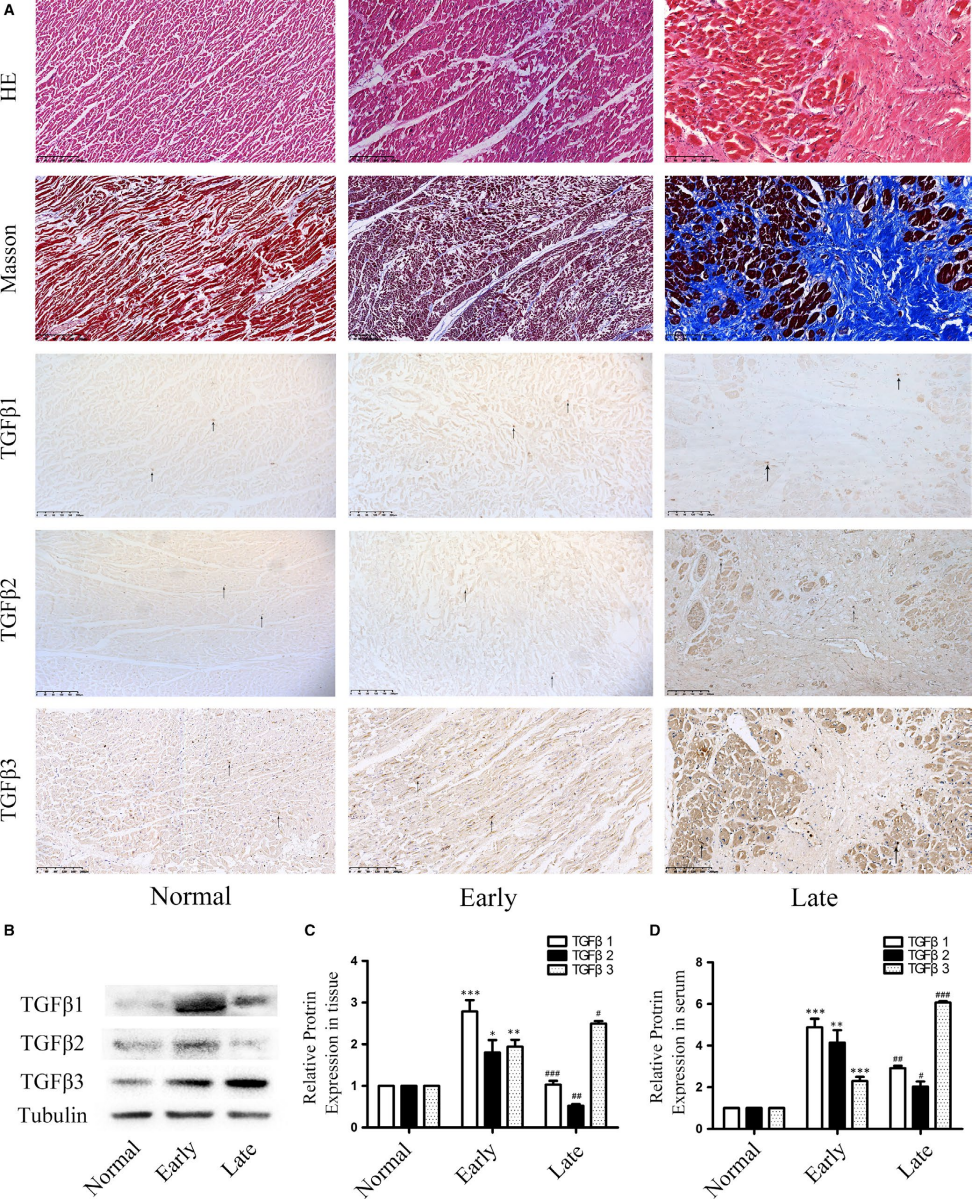
A, The HE staining showed that the coagulative necrosis of cardiomyocytes indicated the early phase of MI and the appearance of collagens indicated the late phase of MI. In Masson staining, the silk‐like fibres indicated the early phase of MI and the appearance of collagen fibres indicated the late phase of MI. The immunohistochemical staining showed the qualitative analysis of TGFβ1, TGFβ2 and TGFβ3 in the normal and MI tissues (n = 6, each group). B,C, Expression levels of TGFβ1, TGFβ2 and TGFβ3 in human tissue after heart infarction according to Western blot and semiquantitative analysis. The results are shown using tubulin as an endogenous control. The data are presented as the mean ± SEM (n = 6, each group). *P < 0.05 versus the normal group; **P < 0.01 versus the normal group; ***P < 0.005 versus the normal group; #*P* < 0.05 versus the early group; ##*P* < 0.01 versus the early group; ###*P* < 0.005 versus the early group. D, TGFβ1, TGFβ2 and TGFβ3 expression levels in human serum after myocardial infarction according to ELISA. The data are presented as the mean ± SEM (n = 6, each group). *P < 0.05 versus the normal group;**P < 0.01 versus the normal group; ***P < 0.005 versus the normal group; #*P* < 0.05 versus the early group; ##*P* < 0.01 versus the early group; ###*P* < 0.005 versus the early group

We evaluated the expression of TGFβ1, TGFβ2 and TGFβ3 in human MI samples and normal samples. HE and Masson staining were performed to group the early and the late phase of MI (Figure S1). HE staining showed the infarction region, while the Masson staining showed that collagen fibres increased with the development of fibrosis (Figure 1A). The immunohistochemical staining for TGFβ1, TGFβ2 and TGFβ3 was quantitatively analysed (Figure 1A). As determined by Western blot and ELISA, relatively high expression of TGFβ1 and TGFβ2 was detected in the early phase of MI, while TGFβ1 and TGFβ2 expressions decreased with the infarction time. Interestingly TGFβ3 was detected increasing gradually with the infarction time and reached the peak about one month after MI, which was different from TGFβ1 and TGFβ2, revealing a distinct effect of TGFβ3 in the development of fibrosis post‐MI (Figure 1B‐D).

### 3.2 TGFβ3 alters the biological behaviours of human CFs

**Figure 2:**
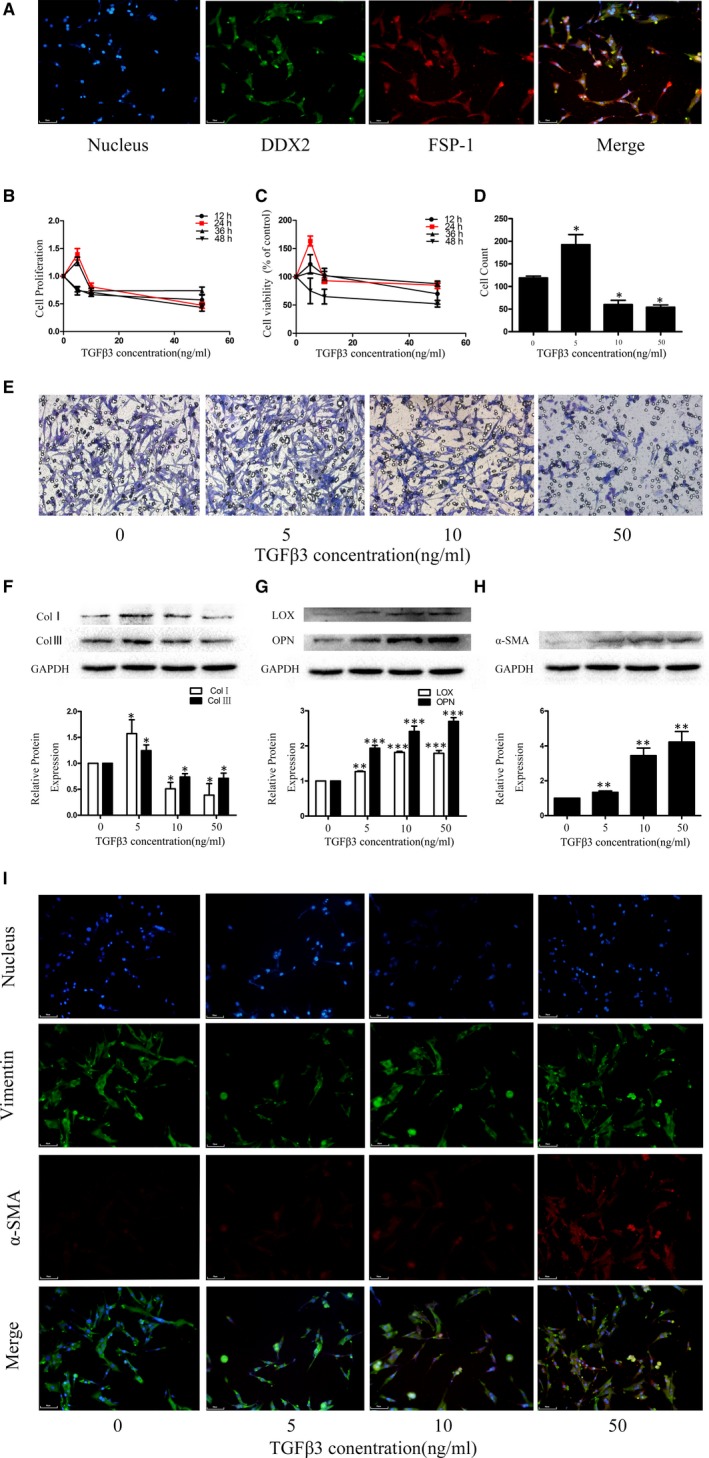
A, An inverted optical microscope was used to visualize human CFs growth adherence to the wall and scattering for most cells. Unlike myocardial cells, the CFs were not clustered and had no spontaneous pulsation. The cells were identified by immunofluorescence, and the results showed that more than 90% of the cells with positive DDX2 and FSP‐1 expression were cultured. Effect of TGFβ3 on cell migration, proliferation and collagen synthesis: B, CCK‐8 assay was applied to evaluate the effects of different concentrations and incubation times of rTGFβ3 on proliferation in human CFs. C, MTT assay was applied to evaluate the effects of different concentrations and incubation times of rTGFβ3 on cytotoxicity in human CFs. D, E, Transwell assays were applied to evaluate the effects of different concentrations of rTGFβ3 on migration in human CFs.*P < 0.05 versus the 0 ng/mL rTGFβ3 group. F, G: Western blot and semiquantitative analyses for collagen I, collagen III, LOX and OPN expression in human CFs treated with rTGFβ3. The results are shown using GAPDH as an endogenous control. The data are presented as the mean ± SEM. *P < 0.05 versus the 0 ng/mL rTGFβ3 group; **P < 0.01 versus the 0 ng/mL rTGFβ3 group; ***P < 0.005 versus the 0 ng/mL rTGFβ3 group; H,I, Western blot, immunofluorescence and semiquantitative analyses for α‐SMA and vimentin. The results are shown using GAPDH as an endogenous control. The data are presented as the mean ± SEM. **P < 0.01 versus the 0 ng/mL rTGFβ3 group

An immunofluorescence assay was performed to confirm the cell line in which DDR2 and FSP‐1 were highly expressed (Figure 2A). Different rTGFβ3 protein concentrations (0, 5, 10 and 50 ng/mL) were used to treat human CFs for 24 hours in this study. The results revealed that compared to the control group, TGFβ3 at a concentration of 5 ng/mL significantly increased the proliferation and migration rate of human CFs and the expression of collagen Ⅰ and collagen III in human CFs; moreover, TGFβ3 at 10 and 50 ng/mL significantly attenuated the proliferation rate and migration rate of human CFs and inhibited the expression of collagen Ⅰ and collagen Ⅲ in human CFs (*P* < 0.05; Figure 2B,D‐F). MTT assay was performed to detect the cytotoxicity of rTGFβ3, and no significant cytotoxic effect was observed (Figure 2C). The results of Western blot showed that TGFβ3 significantly increased the expression of LOX, OPN and α‐SMA gradually, indicating that TGFβ3 induced collagen cross‐linking and phenotypic shift (*P* < 0.05; Figure 2G‐I).

### 3.3 Regulation of the TGFβ3 gene by siRNA/pEX4 altered TGFβ3 expression in human CFs

**Figure 3:**
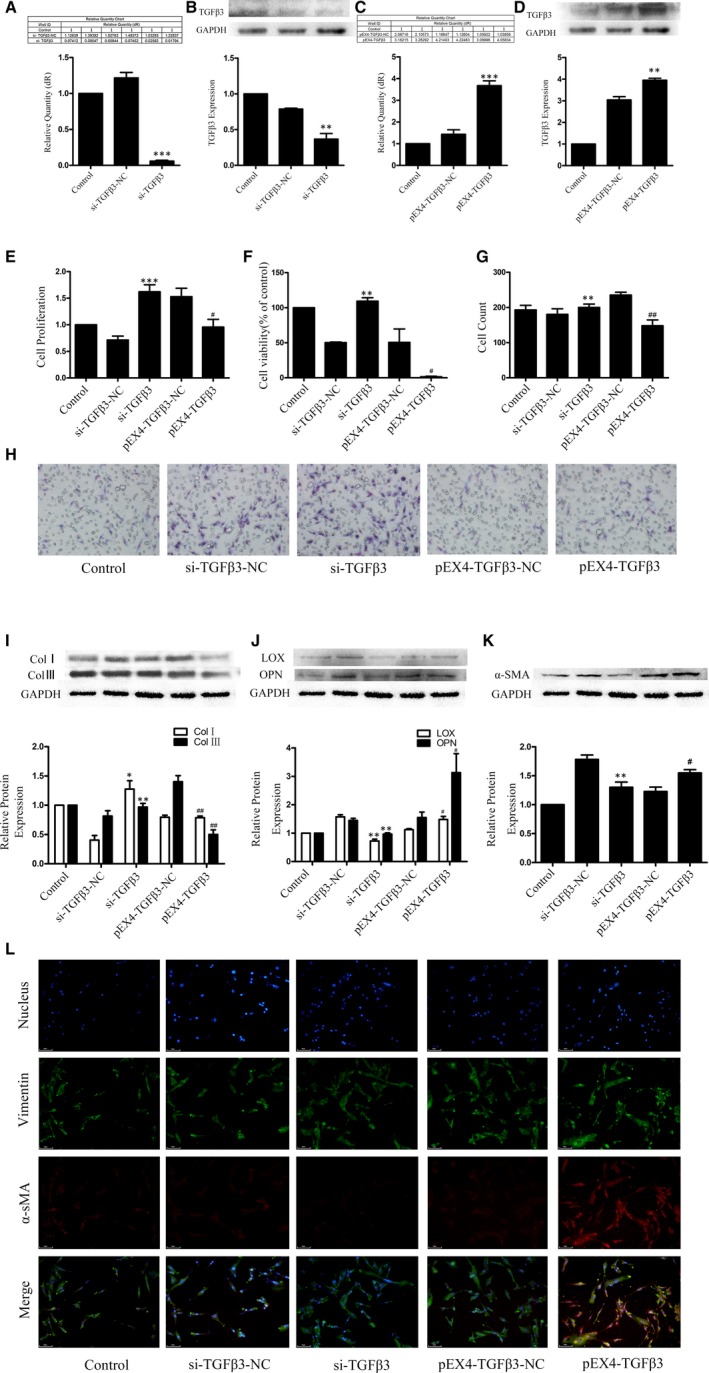
A, B, The transfection efficiency of TGFβ3 siRNA was confirmed by RT‐qPCR, Western blot assays and semiquantitative analyses. The results are shown using GAPDH as an endogenous control. The data are presented as the mean ± SEM. **P < 0.01 versus the si‐TGFβ3‐NC group; ***P < 0.005 versus the si‐TGFβ3‐NC group; C, D, The transfection efficiency of the TGFβ3 plasmid was confirmed by RT‐qPCR, Western blot assays and semiquantitative analyses. The results are shown using GAPDH as an endogenous control. The data are presented as the mean ± SEM. **P < 0.01 versus the pEX4‐TGFβ3‐NC group; *** *P* < 0.005 versus the pEX4‐TGFβ3‐NC group; E, CCK‐8 assay was applied to evaluate of endogenously expressed TGFβ3 on the proliferation of human CFs. ***P < 0.005 versus the si‐TGFβ3‐NC group; # *P* < 0.05 versus the pEX4‐TGFβ3‐NC group. F, MTT assay was applied to evaluate of endogenously expressed TGFβ3 on the cytotoxicity of human CFs. **P < 0.01 versus the si‐TGFβ3‐NC group; # *P* < 0.05 versus the pEX4‐TGFβ3‐NC group. G,H, Transwell assay was applied to evaluate of endogenously expressed TGFβ3 on the migration of human CFs. **P < 0.01 versus the si‐TGFβ3‐NC group; ##*P* < 0.01 versus the pEX4‐TGFβ3‐NC group; I,J, Western blot analyses for collagen I and III, LOX and OPN expression in human CFs with endogenous TGFβ3. The results are shown using GAPDH as an endogenous control. The data are presented as the mean ± SEM. *P < 0.05 versus the si‐TGFβ3‐NC group; **P < 0.01 versus the si‐TGFβ3‐NC group; # *P* < 0.05 versus the pEX4‐TGFβ3‐NC group. ## *P* < 0.01 versus the pEX4‐TGFβ3‐NC group. K,L, Western blot, immunofluorescence and semiquantitative analyses for α‐SMA and vimentin. The results are shown using GAPDH as an endogenous control. The data are presented as the mean ± SEM. **P < 0.01 versus the si‐TGFβ3‐NC group; # *P* < 0.05 versus the pEX4‐TGFβ3‐NC group

To evaluate the functional roles of TGFβ3 in MI, TGFβ3 was knocked down with si‐TGFβ3 (siRNA‐TGFβ3‐homo‐1423) and overexpressed with pEX4‐TGFβ3 transfection in human CFs, the efficiency of which was demonstrated using RT‐qPCR and Western blot assays. The results indicated that the expression of TGFβ3 was successfully inhibited in the si‐TGFβ3 group and overexpressed in the pEX4‐TGFβ3 group compared to that in the control group (*P* < 0.05, Figure 3A‐D).

### 3.4 Endogenous TGFβ3 attenuated the proliferation and migration rates, phenotype shift and collagen expression and promoted LOX and OPN expression

We tested the effects of TGFβ3 siRNA‐mediated knockdown and TGFβ3 pEX4‐mediated over‐expression on human CFs proliferation, migration, collagen synthesis and cross‐linking. Human CFs proliferation and migration were found enhanced in 24 hours, and collagen synthesis found enhanced in 48 hours after the transfection of si‐TGFβ3, while human CFs proliferation and migration were found attenuated in 24 hours, and collagen synthesis found attenuated in 48 hours after the transfection of TGFβ3 pEX4‐TGFβ3 (*P* < 0.05; Figure 3E,G‐I). MTT assay was performed to detect the cytotoxicity of rTGFβ3, and no significant cytotoxic effect was observed (Figure 3F). These data suggested a potential protective role for TGFβ3 in down‐regulating myocardial fibrosis. In addition, the expression of LOX, OPN and α‐SMA was enhanced by TGFβ3 via pEX4‐TGFβ3 and inhibited via siRNA‐TGFβ3 in 48 hours after the corresponding transfections (*P* < 0.05; Figure 3F,J‐L), which confirmed the pivotal role of TGFβ3 in the myocardial remodelling.

### 3.5 TGFβ3 inhibited collagen synthesis and regulated the phosphorylation of TGFβ/smad signalling in Ang II‐induced myocardial fibrosis

**Figure 4:**
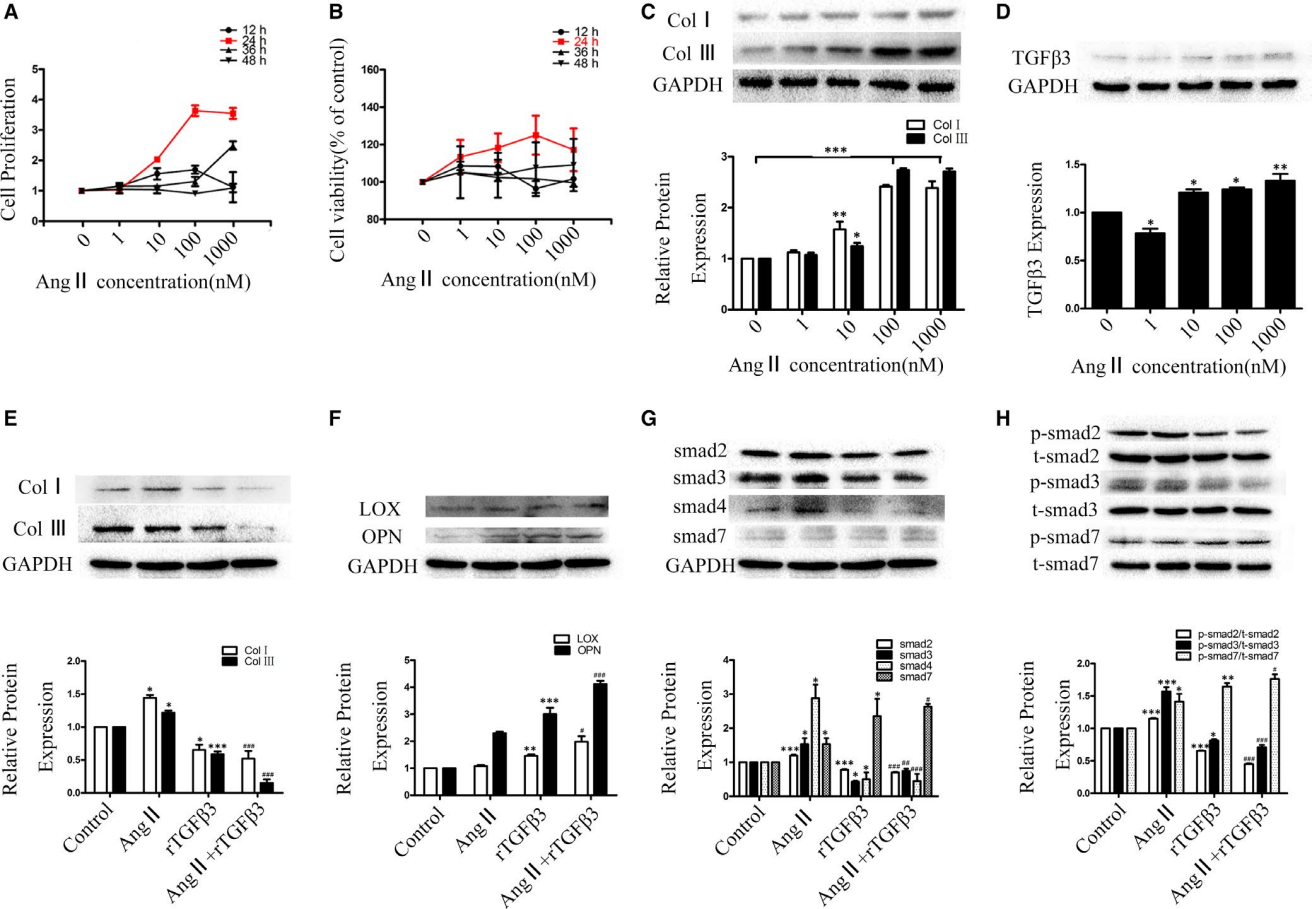
A, B, CCK‐8 and MTT assays were applied to evaluate the proliferation of Ang II‐induced human CFs. The data are presented as the mean ± SEM. C, Western blot and semiquantitative analyses for collagen Ⅰ and Ⅲ, expressions in Ang Ⅱ‐conditioned human CFs. The results are shown using GAPDH as an endogenous control. The data are presented as the mean ± SEM. *P < 0.05 versus the 0 nmol/L group; **P < 0.01 versus the 0 nmol/L group; ***P < 0.005 versus the 0 nmol/L group; D, Western blot and semiquantitative analyses showing the expression levels of TGFβ3 in human CFs at different concentrations of Ang II. The results are shown using GAPDH as an endogenous control. The data are presented as the mean ± SEM. *P < 0.05 versus the 0 nmol/L group; **P < 0.01 versus the 0 nmol/L group. E,F, Western blot and semiquantitative analyses for collagen Ⅰ, collagen Ⅲ, LOX and OPN expression in Ang Ⅱ (100 nmol/L) and TGFβ3 (10 ng/mL) treatment for 24 hours in human CFs. The results are shown using GAPDH as an endogenous control. The data are presented as the mean ± SEM. *P < 0.05 versus the control group; **P < 0.01 versus the control group; ***P < 0.005 versus the control group; #*P* < 0.05 versus the Ang Ⅱ group; ###*P* < 0.005 versus the Ang Ⅱ group; G, H, Effect of p; 24‐h‐Ang Ⅱ (100 nmol/L)‐TGFβ3 (10 ng/mL) treatment on the TGFβ/smad signalling pathway and the relative protein expression. Western blot and semiquantitative analyses for t‐smad2, t‐smad3, smad4, t‐smad7, p‐smad2, p‐smad3 and p‐smad7 expression in human CFs using GAPDH and total smad as endogenous controls. The data are presented as the mean ± SEM.*P < 0.05 versus the control group; **P < 0.01 versus the control group; ***P < 0.005 versus the control group; #*P* < 0.05 versus the Ang Ⅱ group; ##*P* < 0.01 versus the Ang Ⅱ group; ###*P* < 0.005 versus the Ang Ⅱ group

Ang II promotes apoptosis, hypertrophy, CFs proliferation and ECM synthesis and secretion by binding to the angiotensin receptor (ATR), ultimately leading to myocardial remodelling.^18^, ^19^ CCK‐8 and MTT assays indicated that the optimal concentration and stimulation time of Ang II were 100 nmol/L and 24 hours (*P* < 0.05; Figure 4A,B). The Western blot assay indicated that the expression of collagens was significantly higher in the Ang II‐treated group than in the 0 nmol/L group (*P* < 0.05; Figure 4C). Thus, we treated human CFs with Ang II‐induced medium (100 nmol/L) for 24 hours to determine the relationship between myocardial fibrosis and TGFβ3. The Western blot assays indicated that the expression of TGFβ3 was significantly higher in the Ang II‐treated group than in the 0 nmol/L group (*P* < 0.05; Figure 4C,D). These results indicated that the promotion of MI could up‐regulate the expression of TGFβ3, which might be correlated with post‐MI fibrosis (*P* < 0.05; Figure 4D). To study the roles of TGFβ3 (10 ng/mL, 24 hours) in collagen synthesis, cross‐linking and the mechanism of TGFβ3 signal transduction in CFs, we analysed collagen, LOX, OPN expression and the relative levels of TGFβ/smad molecules and their phosphorylation in human CFs. Western blot analyses in which the antibodies were diluted at 1:1000 showed that collagen, smad2, smad3 and smad4 expression and smad2, smad3 phosphorylation levels were lower in the rTGFβ3 and Ang II + rTGFβ3 groups than in the control and the Ang II groups. In addition, smad7 expression and smad7 phosphorylation levels were markedly higher in the rTGFβ3 and Ang II + rTGFβ3 groups than in the control and Ang II groups (*P* < 0.05; Figure 4E,G,H). Western blot analyses showed that LOX and OPN expression levels were higher in the rTGFβ3 and in the Ang II + rTGFβ3 groups than in the control and in the Ang II groups (*P* < 0.05; Figure 4F). Importantly, without serine phosphorylation motif site in its structure smad4 cannot be phosphorylated,^21^ so the phosphorylated smad4 was not evaluated.

### 3.6 TGFβ3 promoted the expression of smad7

**Figure 5:**
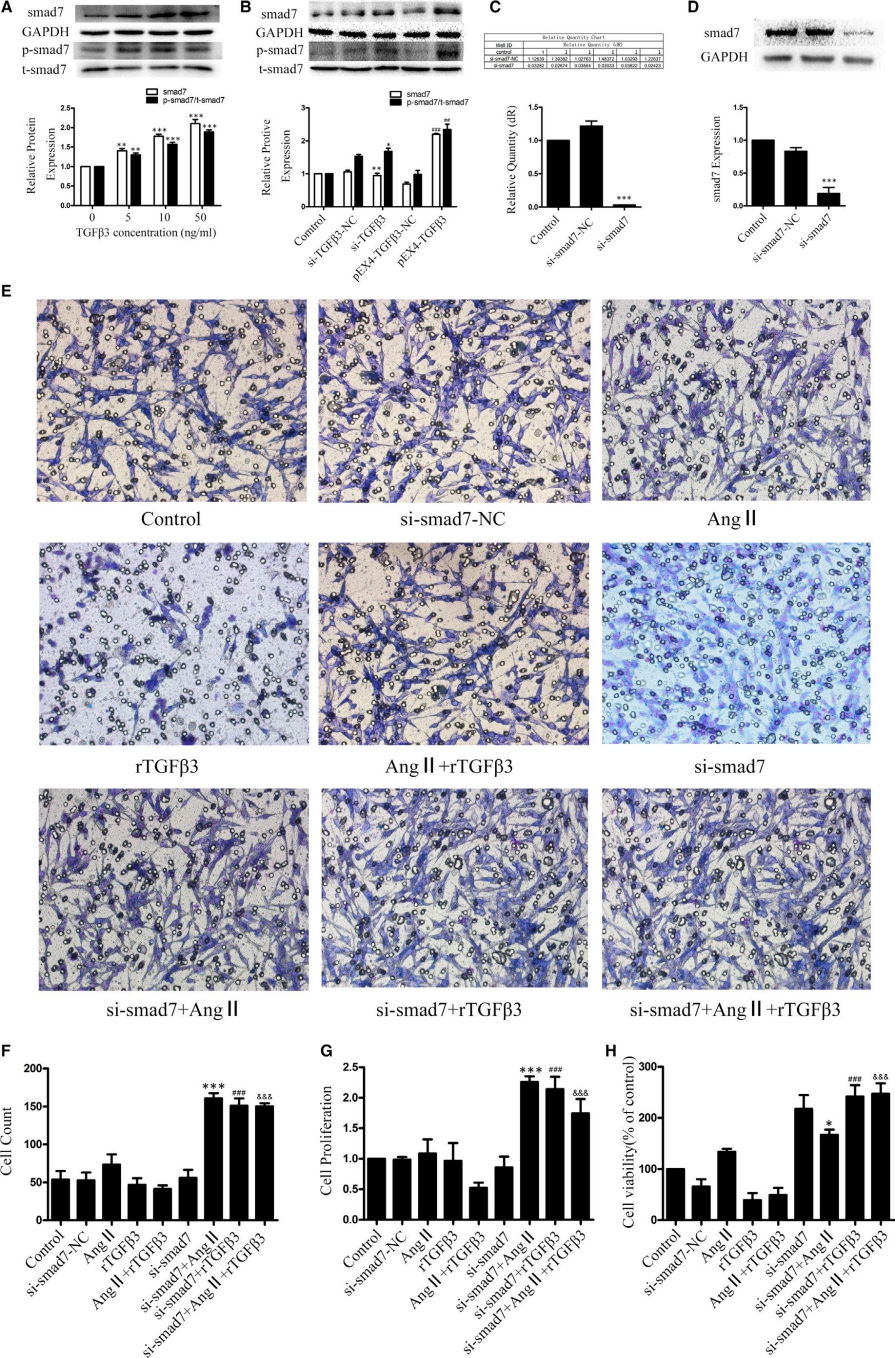
A, Western blot and semiquantitative analyses for smad7 and p‐smad7 expression in human CFs treated with different concentrations of rTGFβ3. GAPDH and total smad were used as an endogenous control. The data are presented as the mean ± SEM. **P < 0.01 versus the 0 ng/mL rTGFβ3 group; *P < 0.005 versus the 0 ng/mL rTGFβ3 group; B, Western blot and semiquantitative analyses for smad7 and p‐smad7 expression in human CFs treated with endogenous TGFβ3. GAPDH and total smad were used as an endogenous control. The data are presented as the mean ± SEM. *P < 0.05 versus the si‐TGFβ3‐NC group; **P < 0.051 versus the si‐TGFβ3‐NC group; ##*P* < 0.01 versus the pEX4‐TGFβ3‐NC group; ###*P* < 0.005 versus the pEX4‐TGFβ3‐NC group; C, The transfection efficiency of smad7 siRNA was confirmed by RT‐qPCR. The results are shown using GAPDH as an endogenous control. ***P < 0.005 versus the si‐smad7‐NC group; D, The transfection efficiency of smad7 siRNA was confirmed by Western blot and semiquantitative analyses. The results are shown using GAPDH as an endogenous control. The data are presented as the mean ± SEM. ***P < 0.005 versus the si‐smad7‐NC group; E, F, Transwell assay and cell count analysis were used on 24‐h‐Ang Ⅱ (100 nmol/L)‐TGFβ3 (10 ng/mL) treatment and 48‐h‐si‐smad7 transfection cells to evaluate the migration effect of smad7 on human CFs. ***P < 0.005 versus the Ang Ⅱ group; ###*P* < 0.005 versus the rTGFβ3 group; &&& *P* < 0.005 versus the Ang Ⅱ+rTGFβ3 group. G, CCK‐8 assay was used on 24‐h‐Ang Ⅱ (100 nmol/L)‐TGFβ3 (10 ng/mL) treatment and 48‐h‐si‐smad7 transfection cells to evaluate the proliferation effect of smad7 on human CFs. ***P < 0.005 versus the Ang Ⅱ group; ###*P* < 0.005 versus the rTGFβ3 group; &&& *P* < 0.005 versus the Ang Ⅱ+rTGFβ3 group. H, MTT assay was used on 24‐h‐Ang Ⅱ (100 nmol/L)‐TGFβ3 (10 ng/mL) treatment and 48‐h‐si‐smad7 transfection cells to evaluate the cytotoxicity effect of smad7 on human CFs. *P < 0.05 versus the Ang Ⅱ group; ###*P* < 0.005 versus the rTGFβ3 group; &&& *P* < 0.005 versus the Ang Ⅱ+rTGFβ3 group

To analyse the effects of TGFβ3 on the expression and phosphorylation levels of smad7, human CFs were pretreated with different concentrations of TGFβ3 for 24 hours. The results revealed that compared to control group, TGFβ3 significantly increased the expressions of smad7 and p‐smad7 (*P* < 0.05; Figure 5A). Western blot analyses showed that the smad7 expression and phosphorylation levels were attenuated in the si‐TGFβ3 groups, while smad7 and p‐smad7 expression levels were increased in 48 hours after the transfection of pEX4‐TGFβ3 (*P* < 0.05; Figure 5B). These results confirmed the pivotal role of smad7 in the effect of TGFβ3 against myocardial fibrosis.

### 3.7 Silencing of the smad7 gene by siRNA inhibited smad7 expression in human CFs

Smad7 siRNA was transfected in human CFs. RT‐qPCR and Western blot assays were used to determine the silencing efficiency. The results showed that si‐smad7(si‐smad7‐homo‐824) successfully attenuated the expression of smad7 (*P* < 0.05, Figure 5C,D).

### 3.8 Smad7 inhibits the proliferation and migration of human CFs induced by Ang II

Human CFs proliferation and migration play an important role in myocardial fibrosis. We treated human CFs with si‐smad7 for 24 hours, then Ang II (100 nmol/L) and rTGFβ3 (10 ng/mL) for 24 hours to determine the relationship between TGFβ3 and samd7 in post‐MI. Transwell migration, CCK‐8 assays were used to evaluate the effects of si‐smad7 on human CFs migration and proliferation. The results indicated that the cell migration and proliferation rates were significantly higher in the si‐smad7‐Ang II‐treated group, the si‐samd7‐TGFβ3 group and the si‐smad7‐Ang II + rTGFβ3 group than the Ang II‐treated group, rTGFβ3 group, Ang II + rTGFβ3 group respectively. MTT assay was performed to detect the cytotoxicity of si‐smad7, Ang II and rTGFβ3, and no significant cytotoxic effect was observed. These results confirmed the pivotal role of smad7 in the certain effect of TGFβ3 against human CFs proliferation and migration (*P* < 0.05, Figure 5E‐H).

### 3.9 TGFβ3 affected Ang II‐induced myocardial fibrosis via TGFβ/smad signalling, which might be attributed to smad7 regulation

**Figure 6:**
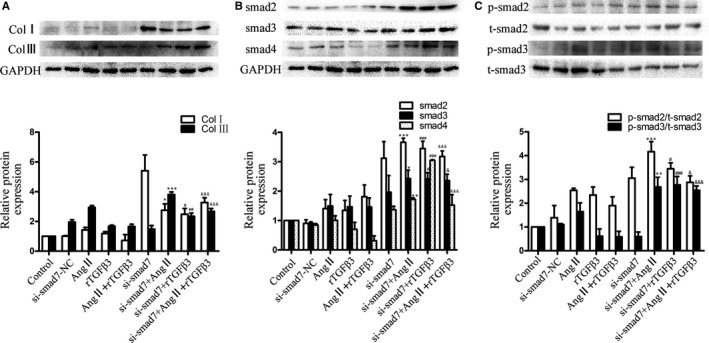
A,B,C, Western blot and semiquantitative analyses for collagen Ⅰ, collagen Ⅲ, t‐smad2, p‐smad2, t‐smad3, p‐smad3 and smad4 expression in human CFs treated with 24‐h‐Ang Ⅱ (100 nmol/L)‐TGFβ3 (10 ng/mL) treatment and 48‐h‐si‐smad7 transfection cells. GAPDH and total smad were used as endogenous controls. The data are presented as the mean ± SEM. *P < 0.05 versus the Ang Ⅱ group; **P < 0.01 versus the Ang Ⅱ group; ***P < 0.005 versus the Ang Ⅱ group; #*P* < 0.05 versus the rTGFβ3 group; ##*P* < 0.01 versus the rTGFβ3 group; ###*P* < 0.005 versus the rTGFβ3 group; & *P* < 0.05 versus the Ang Ⅱ+rTGFβ3 group; &&& *P* < 0.005 versus the Ang Ⅱ+rTGFβ3 group

We performed Western blot to examine the role of smad7 in the TGFβ/smad signalling pathway, which was up‐regulated by TGFβ3 as mentioned previously (Figures 4G,H and A,B). These data confirmed the pivotal role of smad7 in the protective effect of TGFβ3 against myocardial fibrosis. We treated human CFs with si‐smad7 for 24 hours, then Ang II (100 nmol/L) and rTGFβ3 (10 ng/mL) for another 24 hours to determine the effect of smad7 on Ang II‐induced collagen synthesis. When the antibodies were diluted at 1:2000, the expression of collagen Ⅰ, collagen III, smad2, smad3 and smad4 and the phosphorylation levels of smad2 and smad3 were higher in the si‐smad7‐Ang II‐treated group, the si‐smad7‐rTGFβ3 group and the si‐smad7‐Ang II + rTGFβ3 group than the Ang II‐treated group, rTGFβ3 group, Ang II + rTGFβ3 group respectively (*P* < 0.05 Figure 6A‐C). These results indicated that TGFβ3 affected Ang II‐induced myocardial fibrosis via TGFβ/smad signalling, in which smad7 might be involved.

## 4 DISCUSSION

After MI, the infarction area is dominated by inflammatory cells that release cytokines to induce CFs migration to the infarcted area and collagen synthesis and secretion to replace necrotic myocardial tissue.^22^, ^23^ Studies have shown that after MI, CFs undergo phenotypic transformation and acquire a myofibroblast phenotype, which express α‐SMA, for both fibroblasts and smooth muscle cells to maintain the function of the damaged myocardium.^5^ This study focused on understanding the role of TGFβ3 in post‐MI. Here, we found that (a) human MI samples had the higher levels of TGFβ3 than normal samples; (b) TGFβ3 inhibited the migration, proliferation of human CFs, and certain concentrations of TGFβ3 attenuated collagen synthesis and the related protein expressions in human CFs; (c) TGFβ3 promoted the phenotype shift of human CFs and collagen cross‐linking; (d) TGFβ3 affected myocardial fibrosis via TGFβ/smad signalling, in which smad7 might be involved. The TGFβ superfamily includes TGFβs, activins, inhibins, bone morphogenetic proteins (BMPs), Nodal, growth differentiation factors (GDFs), and anti‐mullerian hormone (MIS).^7^ Although the role of other TGFβ superfamily members in pathological fibrosis is still being investigated, the TGFβ subfamily, consisting of TGFβ1, 2 and 3, has long been known to play critical roles in pathological fibrosis. A large number of studies showed that TGFβ1 and TGFβ2 are associated with pathological fibrosis.^24^, ^25^ Unlike TGFβ1 or TGFβ2, TGFβ3 appears to be a very promising inhibitor of pathological fibrosis in the skin,^26^ vocal fold mucosa,^27^ cornea 28 and lungs.^29^ In our study, relatively higher expression levels of TGFβ1, TGFβ2 and TGFβ3 were detected in the infarcted samples than in the control samples. We found higher expression of TGFβ3 was detected in the late stage than the early. In addition, the relevant literature on animal experiments showed the peak of TGFβ3 was reached in the day 32 after MI.^11^ Similarly, we found that TGFβ3 was secreted gradually and reached the peak in one month after MI. In contrast, TGFβ1 and TGFβ2 expression showed the opposite trend. A study on post‐burn scarring showed TGFβ1 levels significantly increased during the first 2 weeks post‐injury and then decreased,^30^ while the scarless wound healing observed persistent increased levels of TGFβ3.^10^ Consequently, the complicated changes in the ratio of TGFβ isoforms will lead to tissue remodelling. Thus, insight into TGFβ3 is necessary to determine the relationship among the isoforms of TGFβ.

CFs play a critical role in maintaining ECM homeostasis in response to pathological stimuli. It has been shown that DDR2,^31^ FSP‐1 32 and vimentin, which we detected, have been proposed to be the markers of cardiac fibroblasts and fibroblasts. These cells have numerous functions, including ECM synthesis and deposition, cell‐to‐cell communication and signalling with myocytes, other CFs and endothelial cells. In particular, the biological behaviours of CFs become dysregulated during myocardial remodelling. Fibrosis is mainly characterized by migration and proliferation of CFs.^33^, ^34^ It had been reported that TGFβ3 could attenuate CFs proliferation and differentiation in skin.^36^ In addition, Simon N and Waddington, et al provided strong evidence that the presence of mutTGFβ3 at the wound site significantly reduced the amount of CFs, retarded CFs migration and attenuated collagen synthesis within the wound area.^9^ In this study, we found that TGFβ3 increased migration, proliferation and collagen synthesis at 5 ng/mL and attenuated migration, proliferation and collagen synthesis at 10 and 50 ng/mL. It was reported that collagen synthesis was stimulated in response to TGFβ3 through TGFβ1‐dependent and TGFβ1‐independent manners,^37^ which indicated that the increasing levels of TGFβ1 could be a result of the increasing levels of TGFβ3. As we showed before, TGFβ3 increased gradually with the infarction time, when the ratio of TGFβ1 to TGFβ3 changed, the inhibition of TGFβ3 predominated and the whole body experienced the inhibition effect.

It was reported that fibroblasts disappeared at the end of the fibroproliferative phase in normal wound healing,^38^ which suggested that TGFβ3 played a dominant role in the fibrous phase of myocardial remodelling. At this part of the maturation phase, CFs have acquired the capacity to remodel the ECM via activated integrins and cadherin receptor proteins.^39^ They produce high levels of procollagen fibrils which are cross‐linked by LOX,^40^ and LOX was activated by OPN.^41^ In the setting of MI, a subset of activated cardiac fibroblasts acquires new phenotypic characteristics, including the expression of the contractile protein α‐SMA contributing to pathological cardiac remodelling.^5^ In this study, we found that the expression of LOX, OPN and α‐SMA increased with increasing concentrations of TGFβ3 continuously, which showed different results of collagens. In addition, LOX, OPN and α‐SMA were enhanced by pEX4‐TGFβ3, and attenuated by si‐TGFβ3. Taken together, these indicated that TGFβ3 could promote myocardial remodelling.

Thus, TGFβ3 might mediate the attenuating effect on CFs proliferation, ECM production and myocardial fibrosis, and promotion effect on collagen cross‐linking, which might provide a new understanding and TGFβ3‐based therapeutic strategy for myocardial remodelling. Fibrosis, the leading cause of myocardial remodelling, is correlated with the deposition of excess ECM proteins, such as collagens. Collagen homeostasis plays a key role in myocardial fibrosis. It has been reported that TGFβ3 played a protective role in pancreatic fibrosis^42^ and hepatic fibrosis^43^ by attenuating the expression of collagen Ⅰ and Ⅲ. It is well known that Ang II is a potent and key profibrotic factor in CFs proliferation, migration, collagen synthesis and cross‐linking.^44^ Meanwhile, Ang II, which increased with infarction timing,^45^ significantly promoted TGFβ3 expression. Based on these results, we further confirmed TGFβ3 increased gradually with the infarction time. Importantly, it was indicated that the promotion of MI could up‐regulate the expression of TGFβ3, which might be correlated with post‐MI fibrosis. We examined whether TGFβ3 regulated collagen I, III, LOX and OPN synthesis after MI. The results exhibited attenuated collagen I and collagen III expression in the Ang II + rTGFβ3 groups. While increased expressions of LOX and OPN were detected in the Ang II groups, rTGFβ3 groups and Ang II + rTGFβ3 groups. Therefore, we speculated that TGFβ3 might have protective effects against Ang II‐induced myocardial fibrosis.

TGFβ signalling is mediated mainly by a pair of transmembrane serine‐threonine kinase receptors called TβRI (or activin receptor‐like kinase‐5, ALK5) and TβRII. TGFβ receptors propagate TGFβ signal transduction in humans through the recruitment of smads, including smad2, smad3, smad4 and smad7; smad2 and smad3 are known as activation smads, and smad4 is known as a common smad, which plays an essential role in fibrosis diseases by enhancing smad3‐responsive promoter activity. TGFβ ligands combine TβRI and TβRII to phosphorylate smad2 and smad3. Once activated by TGFβ receptors, phosphorylated smad2 and smad3 form a complex with smad4 and translocate to the nucleus, where these proteins function as transcription factors alone or in association with other DNA binding factors to modulate target gene expression.^46^ The TGFβ/smad signalling pathway may represent an important regulatory target that controls myocardial remodelling. Therapeutic agents that target the TGFβ/smad signalling pathway to reduce scarring have been successful in pre‐clinical studies.^7^ Inhibitors of the TGFβ receptor ALK5 decreased TGFβ activity, rescued cardiac dysfunction and ameliorated the remodelling that occurs post‐MI.^47^ In a bleomycin‐lung fibrosis model, the fibrotic process was attenuated in mice lacking smad3.^48^, ^49^ We examined the attenuated expressions of collagen I, collagen III, smad2, p‐smad2, smad3, p‐smad3 and smad4 in the Ang II + rTGFβ3 groups. Therefore, it was confirmed that TGFβ3 could attenuate the synthesis of collagens post‐MI by reducing the phosphorylation of the TGFβ/smad signalling pathway.

Smad7 is known as an inhibitory smad (I‐smad) or a protective smad that switches off the TGFβ/smad signalling pathway.^50^ In the context of hepatic fibrosis, smad7 negatively mediates smad3‐induced fibrogenesis.^51^ Earlier reports have shown that TGFβ3 may regulate smad7 proteins through the TGFβ/smad signalling pathway to minimize extrinsic scarring 52 and hepatic fibrosis.^53^ Our main interest was to investigate the relationship between TGFβ3 and smad7 activation in MI. Our findings, which showed that the migration and proliferation of human CFs and the expression of collagen I, collagen III, smad2, smad3 and smad4 and the phosphorylation levels of smad2 and smad3 were higher in si‐smad7 groups than in the relevant control groups, indicated an essential role for smad7 in post‐MI cardiac healing.

According to our experimental results, Ang II‐induced cell proliferation and migration were significantly inhibited after the down‐regulation of smad7. So we concluded that smad7, which belonged to the smad superfamily located downstream of the mitogen‐activated protein kinase signalling pathway, normally counteracted the effects of Ang II on cell proliferation and migration. Moreover, the effect produced by silencing smad7 was not reversed by the treatment with TGFβ3, which was expected because smad7 is downstream of the TGFβ3 signalling pathway. Ultimately, we addressed the possibility of novel therapeutic approaches targeting TGFβ3 and smad7 for treating MI‐induced myocardial remodelling.

## 5 CONCLUSION

Our results suggested that TGFβ3 could attenuate the proliferation, migration capability and collagen synthesis of human CFs by modulating the TGFβ/smad signalling pathway, which might be attributed to the regulation of smad7.

## CONFLICT OF INTEREST

The authors confirm that there is no conflict of interest.
