# Supplementary figures and images for: The role and mechanism of transforming growth factor beta 3 in human myocardial infarction‐induced myocardial fibrosis
Source: J Cell Mol Med. 2019 Apr 14;23(6):4229–43. doi: 10.1111/jcmm.14313 (PMC6533491; doi:10.1111/jcmm.14313)

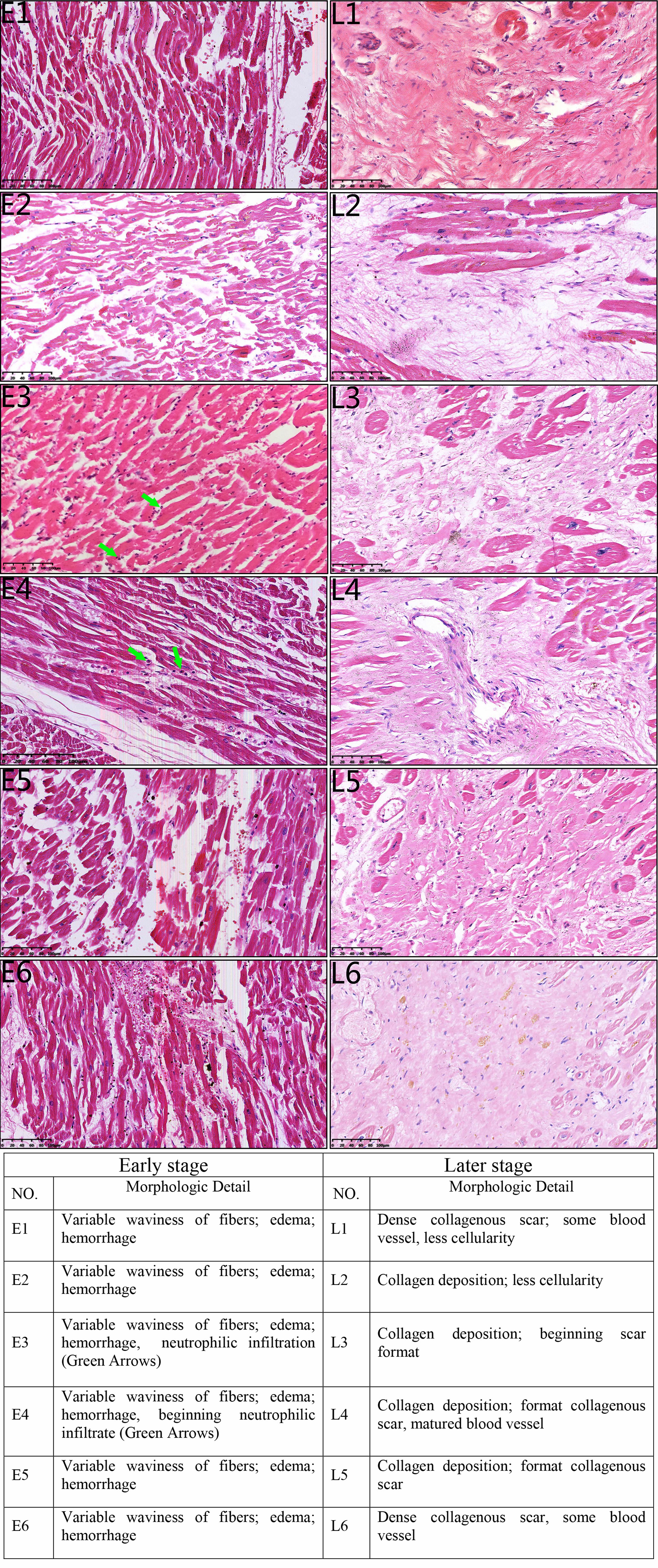

Supplement: Supplementary file 1 [file JCMM-23-4229-s001.jpg]
